# Identification and validation the predictive biomarkers based on risk-adjusted control chart in gemcitabine with or without erlotinib for pancreatic cancer therapy

**DOI:** 10.3389/fgene.2024.1497254

**Published:** 2024-12-17

**Authors:** Aijun Zhao, Dongsheng Tu, Ye He, Liu Liu, Bin Wu, Yixing Ren

**Affiliations:** ^1^ School of Mathematical Science and Geomathematics Key Laboratory of Sichuan Province, Chengdu University of Technology, Chengdu, China; ^2^ Department of Public Health Sciences, Canadian Cancer Trials Group, Queen’s University, Kingston, ON, Canada; ^3^ Visual Computing and Virtual Reality Key Laboratory of Sichuan Province, Sichuan Normal University, Chengdu, China; ^4^ College of Management Science, Chengdu University of Technology, Chengdu, China; ^5^ Department of General Surgery, Institute of Hepato-Biliary-Pancreas and Intestinal Disease, Affiliated Hospital of North Sichuan Medical College, Nanchong, China

**Keywords:** pancreatic cancer, COX proportional hazards regression model, score test, risk-adjusted control chart, predictive biomarkers

## Abstract

**Background:**

In a randomized clinical controlled trial (PA.3) conducted by the Canadian Cancer Trials Group, the effects of gemcitabine combined with the targeted drug erlotinib (GEM-E) *versus* gemcitabine alone (GEM) on patients with unresectable, locally advanced, or metastatic pancreatic cancer were studied. This trial statistically demonstrated that the GEM-E combination therapy moderately improves overall survival (OS) of patients. However, real-world analysis suggested that GEM-E for pancreatic cancer was not more effective than GEM. The heterogeneity in outcomes or treatment effect exist. Thus, we tried to find predictive biomarkers to identifying the heterogeneous patients.

**Methods:**

Of the 569 eligible patients, 480 patients had plasma samples. Univariate and multivariate Cox proportional hazards model were used to identify baseline characteristics related to OS, and a risk adjusted Exponentially Weighted Moving Average (EWMA) control chart based on a weighted score test from the Cox model was constructed to monitor patients’ survival risk. Maximally selected rank statistics were constructed to identifying the predictive biomarkers, in addition, a risk adjusted control chart based on a weighted score test from the Cox model was constructed to validating the predictive biomarkers, discover the patients who sensitive to the GEM-E or GEM.

**Results:**

Three baseline characteristics (ECOG performance status, extent of disease, and pain intensity) were identified related to prognosis. A risk-adjusted EWMA control chart was constructed and showed that GEM-E did improve OS in a few patients. Three biomarkers (BMP2, CXCL6, and HER2) were identified as predictive biomarkers based on maximum selected rank test, and using the risk-adjusted EWMA control chart to validate the reality and discover some patients who are sensitive to the GEM-E therapy.

**Conclusion:**

In reality, GEM-E has not shown a significant advantage over GEM in the treatment of pancreatic cancer. However, we discovered some patients who are sensitive to the GEM-E therapy based on the predictive biomarkers, which suggest that the predictive biomarkers provide ideas for personalized medicine in pancreatic cancer.

## 1 Introduction

Pancreatic ductal adenocarcinoma (PDAC) is a common gastrointestinal malignancy with a 5-year survival rate of less than 10%. Locally advanced and metastatic PDAC accounts for 70%–80% of cases, with only about 10% of patients being eligible for surgical resection (Kung and Yu, 2023). Even with surgical treatment, patients still face high risks of recurrence and metastasis, and these recurrent and advanced patients typically respond poorly to treatment (Neoptolemos et al., 2004). Despite advancements in surgical techniques, the introduction of new drugs, and optimization of comprehensive treatment plans over the past decade, the prognosis for PDAC patients has not significantly improved.

In 1997, gemcitabine was approved by the U.S. Food and Drug Administration for the treatment of PDAC. It not only directly affects tumor cells by inhibiting their growth and division but also alters the tumor microenvironment, making it more sensitive to immunotherapy (Hochster et al., 2006; Burris 3rd et al., 2023). Subsequently, eight phase III trials involving new cytotoxic drugs (Berlin et al., 2002; Rocha Lima et al., 2004; Richards et al., 2004; Moore et al., 2007) or biological agents (Bramhall et al., 2002; Van Cutsem et al., 2004) combined with gemcitabine showed no significant improvement in survival compared to gemcitabine alone. In pancreatic tumors, the epidermal growth factor receptor type 1 (EGFR) is often overexpressed, meaning these signaling pathways are excessively activated, leading to the excessive growth and spread of tumor cells (Fjällskog et al., 2003; Tobita et al., 2003). Erlotinib is an EGFR tyrosine kinase inhibitor that inhibits tumor cell proliferation and survival by blocking the EGFR signaling pathway. The EGFR signaling pathway is also associated with immune suppression in the tumor microenvironment, and blocking this pathway may reduce tumor immune evasion (Ng et al., 2002). In a double-blind, international phase III trial (PA.3) conducted by the Canadian Cancer Trials Group, combination of gemcitabine with erlotinib (GEM-E) in treating patients with unresectable, locally advanced, or metastatic PDAC was studied and compared with GEM. The results showed a small increase in the median survival patients treated by GEM-E (6.24 months *versus* 5.91 months when treated by GEM; Moore et al., 2007). Although this increase was statistically significant and GEM-E was also approved by the U.S. Food and Drug Administration for the treatment of advanced pancreatic cancer in patients who have not received previous chemotherapy based on these results (Kelley and Ko, 2008), it did not significantly outperform monotherapy in terms of objective response rates and was accompanied by mild to moderate side effects. Furthermore, real-world analysis suggested that GEM-E for pancreatic cancer is not more effective than GEM, and it does not provide reasonable cost-effectiveness over GEM (Shin et al., 2016).

Predictive biomarkers help optimize treatment decisions by providing information on the likelihood of response to a given chemotherapy (Ballman, 2015). MSI can serve as a predictive biomarker for fluorouracil treatment in colorectal cancer patients (Ribic et al., 2003). EGFR kinase domain mutations can serve as a predictive biomarker for tyrosine kinase inhibitor treatment in non-small cell lung cancer patients (Riely et al., 2006). The analysis of the HER2 gene amplification is the basic genetic test used in cancer diagnostics for the evaluation of the eligibility of breast cancer patients for treatment with trastuzumab or lapatinib (Mitra et al., 2009). Molecular characterization of CTCs can help predict therapy response. For example, Reinholz et al. showed that decreased mammaglobin 1 (MGB1) mRNA levels in CTCs from metastatic breast cancer patients may predict therapy response (Reinholz et al., 2011). The assessment of the mutation status in codons 12 and 13 of the KRAS gene is a standard predictive biomarker in the evaluation of the eligibility of patients with advanced CRC for targeted therapy using monoclonal antibodies such as cetuximab or panitumumab (Lewandowska et al., 2012). Significant correlations between high TMB and response to immune checkpoint inhibitors have been reported in several cancer types, including urothelial carcinoma, small cell lung cancer, melanoma, and HPV-negative, according to studies (Bai et al., 2020). BRCA1/2 mutations to guide treatment with olaparib in breast cancer (Gennari et al., 2021). For a more detailed review of predictive biomarkers, please refer to the study of Cooper and Tan (2024).

Identifying predictive biomarkers related to PDAC is crucial. Previous studies have validated some biomarkers as being associated with prognosis, which can help predict the PDAC progression and the patient survival. CA19-9 is currently the most commonly used serum marker for PDAC, with high levels of CA19-9 usually associated with poorer prognosis, although it may be falsely elevated in patients with obstructive liver disease or pancreatitis, limiting its specificity and sensitivity (Steinberg, 1990). CEA is another commonly used tumor marker, although its sensitivity and specificity are not as high as CA19-9, high levels of CEA are also associated with poorer prognosis (Kato et al., 2022). In addition, gene mutations or deletions such as KRAS, TP53, and SMAD4 are common in PDAC and are associated with poorer prognosis and higher risk of metastasis (Stefanoudakis et al., 2024). Changes in the expression levels of certain microRNAs (miRs) are associated with PDAC prognosis. For example, high levels of miR-21 and miR-155 are usually associated with poorer prognosis (Barrera et al., 2023). These biomarkers influence the prognosis of PDAC by regulating tumor growth, invasion, and metastasis. Predictive biomarkers may modify the effect of, or have an interaction with a specific treatment, is used to identify the patients with different effects for this treatment. The identification of predictive biomarkers has been an active area of clinical research since it can provide guidance on the best treatment for patients. Shultz et al. (2016) used a multivariable Cox model with an additional interaction term between the treatment (GEM-E vs. GEM) and the binary biomarker expression level (whether it was greater than the median) to find the predictive biomarker. However, the method of splitting the continuous biomarker into the binary biomarker by the median limited them to finding only one predictive biomarker. Hothorn and Zeileis (2008) proposed a predictive biomarker search method to find the optimal cut-point by maximizing the difference between the survival curves of the two groups (whether it was greater than the cut-point), which is employed to search for a high-risk group of rectal cancer patients treated with a neoadjuvant chemoradiotherapy. However, the underlying assumption of the above approach is that treatment effect was invariant for patients in the high-risk or low-risk subgroup, but due to the complexity of the disease, this assumption does not necessarily hold true and there were unobservable factors that may lead to inconsistent.

Statistical process control methods have become increasingly popular in survival analysis due to their advantages in detecting unobserved changes, the purpose of control charts is to promptly detect abnormal changes in process parameters and trigger alarm signals for appropriate action. The most commonly used control chart is the EWMA control chart, which smooths data fluctuations by assigning more weight to recent data points, thereby highlighting potential changes with the small and medium-scale deviations (Lucas and Saccucci, 1990). The control chart operates in two phases. In Phase I, a set of historical observations is used to estimate the baseline hazard, and regression parameters (Jones-Farmer et al., 2014), yielding the chart statistic. Generally, control limits are determined using a given expected false alarm rate. In Phase II, as new observations are collected, the chart statistic is calculated. If the chart statistic is above the upper control limit, the risk of survival increases significantly, and if the chart statistic is below the lower control limit, the risk of survival decreases significantly. If the chart statistic exceeds the control limits, an out-of-control alarm signal is triggered; otherwise, new data continue to be accumulated. To assess patient survival risk, Woodall et al. (2015) adopted a risk-adjusted model incorporating surgical factors to account for heterogeneity at the patient level, and then used chart statistics to monitor adjusted survival risks. Liu et al. (2018) proposed an online weighted score test for binary survival outcomes to detect changes in the mean and variance of postoperative risk. Lai et al. (2021) proposed a risk-adjusted control chart based on a weighted score test for the Cox model to monitor changes in average surgical risk and the existence of its variance, which could be of interest in practical surgical monitoring programs. Therefore, Statistical process control methods help us validate the reality of the predictive biomarker and discover some patients who are sensitive to the GEM-E therapy.

In this study, we utilized a two-sided risk-adjusted control chart to monitor survival risk in PDAC patients after receive GEM-E or GEM therapy. Initially, using the pre-processed PA.3 dataset, univariate and multivariate Cox proportional hazards regression models were employed to identify the baseline characteristics associated with OS. Subsequently, a risk-adjusted control chart based on the weighted score test was constructed to monitor patient survival risk online (Lai et al., 2021) and compare the efficacy of the two treatment regimens (GEM-E and GEM). We identified the predictive biomarkers based on maximizing the rank statistics (Lausen and Schumacher, 1992). Furthermore, A risk-adjusted EWMA control chart based on a weighted score test from the Cox model was constructed to validate the predictive biomarkers, discover the patients who sensitive to the GEM-E or GEM. The study could potentially unveil key predictive biomarkers for personalized medicine of pancreatic cancer.

## 2 Materials and methods

### 2.1 Data source

The Canada Cancer Trials Group (CCTG) PA.3 is an international double-blind phase III clinical trial in which patients were randomized in a 1:1 ratio to receive gemcitabine plus erlotinib or matching placebo after signing a written informed consent form, with the primary endpoint being OS (Moore et al., 2007). In a previous study, the levels of 35 key proteins selected from global genetic analysis of pancreatic cancer were quantified in the plasma of 20uL patients using proximity ligation assays based on a standard protocol including quality control for measurements (Fredriksson et al., 2007; Shultz et al., 2016). Of the 569 eligible patients randomized, 480 had plasma cases prior to treatment, age (years), sex (female vs. male), pain intensity (a scale of 0–100), extent of disease (locally advanced vs. distant metastases), as ECOG performance status (0 or 1) were covariates included in the CCTG clinical database. 15 biomarkers were included in the analysis of this paper from the database (Table 1).

**TABLE 1:** Fifteen biomarkers included in the analysis.

Abbreviation	Full name	Relevant function
CXCL6	Chemokine (C-X-C motif) ligand 6	Promotes chemotaxis of immune cells
CEA	Carcinoembryonic antigen	Often associated with cancer progression and poor prognosis
CA19-9	Carbohydrate associated antigen 19–9	Its level changes may reflect tumor burden
HIF1-alpha	Hypoxia inducible factor 1-alpha	Promotes tumor cell survival under low oxygen conditions
IL6	Interleukin 6	Promotes immune suppression in the tumor microenvironment
IL8	Interleukin 8	Promotes tumor growth, angiogenesis, and metastasis
REG4	Regenerating islet-derived family, member 4	Associated with cell growth, survival, and anti-apoptosis
CXCL9	Chemokine (C-X-C motif) ligand 9	Related to immune cell recruitment and tumor immune response
IGF2	Insulin-like growth factor 2	Promotes tumor cell growth
MMP1	Matric metallopeptidase 1	Degrades extracellular matrix, promoting tumor invasion and metastasis
PF4	Platelet factor 4	Associated with platelet activation and inflammatory response
HER2	erb-b2 receptor tyrosine kinase 2	Highly expressed in certain types of cancer
AXL	AXL receptor tyrosine kinase	Promotes tumor cell survival and immune evasion
BMP2	Bone morphogenetic protein 2	Plays a role in bone formation and the tumor microenvironment
GAS6	Growth arrest specific 1	Involved in cell growth and survival

### 2.2 Statistical methods and analyses

#### 2.2.1 Identification of baseline characteristics significantly affecting patient OS

We removed samples with missing biomarkers as done by Shultz et al. (2016). The final dataset consists of 480 cases, with 246 patients treated with GEM and 234 patients treated with GEM-E. Data are presented as the medians (Med) and interquartile ranges (IQR) for continuous variables and as counts and proportions for categorical variables. Baseline differences between the GEM-E and GEM groups were assessed using the Mann-Whitney test (Wilcoxon Rank test) for continuous variables and the exact Fisher test for categorical variables. Univariate and multivariate Cox proportional hazards regression models were employed to identify baseline characteristics related to OS.

#### 2.2.2 Risk-adjusted control chart based on a weighted score test for monitoring survival risks and comparing efficacy of GEM-E vs. GEM

Since the Cox model does not require any prior distributional assumptions about the data and allows for non-parametric estimation of the baseline hazard function, we used a Cox regression model to adjust for individual patient heterogeneity. We use the Schoenfeld residual test to check whether the assumption holds (Grambsch and Therneau, 1994), the result shown in Supplementary Table S1 of the Supplementary Material A1, the *p*-values were all larger than 0.05, indicating that the assumption of the univariate and multivariate Cox proportional hazards regression model holds. To account for the volatility of survival risk, we incorporated random effects into the Cox regression model to indirectly describe abnormal shifts in the survival risk function: a random effects variance of zero indicates stable efficacy, while a variance greater than zero indicates unstable efficacy. We used a weighted score test statistic derived from homogeneity tests to examine whether the variance of the random effects is significantly zero (Liang, 1987). To effectively detect small to moderate changes in survival risk, an EWMA control chart was constructed using the above-mentioned score test statistic to monitor changes and volatility in patient survival risk online (RAES chart). The expected false alarm rate of the control chart parameters determines the control limits ( $L_{l}$ , $L_{u}$ ) of the chart, distinguishing between common cause variation and special cause variation. In practical applications, the control limits (i.e., the expected false alarm rate) can be adjusted according to specific situations in order to meet different quality control requirements. Given that the sample size of the test set (GEM-E) is 234, the expected false alarm rate was set at 0.5%. This implies that, under controlled conditions, one false alarm (Type I error) is anticipated on average every 200 monitoring instances. This approach strikes a balance between the false alarm rate (Type I error) and the missed detection rate (Type II error) (Lai et al., 2021).

#### 2.2.3 Identification of predictive biomarkers for PDAC using maximum log-rank statistics

We used the “surv_cutpoint” function to select the optimal cut-points for 15 biomarkers based on their expression levels, aiming to maximize the differences between the survival curves of the two groups (i.e., the cut-point that yields the maximum log-rank statistic; Hothorn and Zeileis, 2008). This allowed us to stratify patients into high-risk and low-risk groups, and for each biomarker, we tested its effect in predicting for improved survival due to GEM-E using a multivariable Cox model including an additional interaction term between the treatment (GEM-E vs. GEM) and the binary biomarker expression level (based on whether it was greater than the optimal cut-points). The binary biomarker with a significant interaction term with the treatment was the potentially predictive biomarker.

#### 2.2.4 Monitoring patient survival risk using the RAES chart to identify treatment-sensitive population types

Based on the biomarkers identified in the previous section that could predict the efficacy of specific treatment regimens, we used the RAES chart to monitor the survival risk of PDAC patients. The RAES chart calculated patient survival risk based on cumulative hazard, using PDAC patients treated with GEM therapy as the training set and those treated with GEM-E therapy as the test set. We would analyze the survival risk of patients in both high and low expression cases of these biomarkers. First, we would calculate the baseline cumulative hazard for patients using the training set data. Then, using the test set data, we would calculate the cumulative risk for each case based on patients’ survival outcomes, survival times, and significant influencing factors. Subsequently, we would use the EWMA method to calculate the adjusted survival risk value for each case and plot a line graph showing changes in risk values along the case sequence. The chart would also include horizontal reference lines to visually compare patient risk levels, and indexes would be used to identify cases with survival risk values exceeding a given threshold, thereby identifying populations sensitive to treatment for further analysis of their characteristics.

#### 2.2.5 Statistical analysis

All statistical analyses and plotting would be conducted using R software (version 4.3.1) and RStudio (2023.09.1 + 494). The level of statistical significance was set at *p* or adjusted *p* < 0.05.

## 3 Results

### 3.1 Patient characteristics

Of the 569 eligible patients randomized, 480 had plasma cases prior to treatment, baseline characteristics were well-balanced between the GEM-E and GEM cohorts. As shown in Table 2, there were no significant differences between the two groups in terms of age, sex, ECOG performance status (ECOG), extent of disease (EOD), and pain intensity (PI). Specifically, the median age of the GEM and GEM-E groups were 63.800 (IQR: 55.850–70.775) and 64.100 (IQR: 56.550–71.950), respectively (*p*-value = 0.698), the median PI was 25.215 (IQR: 7.392–48.990) in the GEM group and 23.070 (IQR: 5.950–44.210) in the GEM-E group (*p*-value = 0.205). In terms of sex distribution, 57.3% of the GEM group were male, compared to 49.1% in the GEM-E group (*p*-value = 0.082). Regarding ECOG scores, the majority of patients in both groups had a score of 1, with a *p*-value of 0.728. For EOD, 73.2% of the GEM group and 75.2% of the GEM-E group had distant metastasis (*p*-value = 0.677). Overall, these baseline characteristics showed no significant differences between the two treatment groups, indicating that they were comparable at the start of treatment and providing a solid foundation for subsequent efficacy comparisons.

**TABLE 2:** Baseline Characteristics of the GEM vs. GEM-E cohorts based on 480 patients.

Characteristics	GEM	GEM-E	*p*-value
	(n = 246)	(n = 234)	
Age, Med (IQR)	63.800 (55.850–70.775)	64.100 (56.550–71.950)	0.698
Sex			
1, Male	141 (57.3%)	115 (49.1%)	0.082
0, Female	105 (42.7%)	119 (50.9%)	
ECOG			
1, Limited activity	201 (81.7%)	188 (80.3%)	0.728
0, Fully active	45 (18.3%)	46 (19.7%)	
EOD			
1, Distant metastatic	180 (73.2%)	176 (75.2%)	0.677
0, Locally advanced	66 (26.8%)	58 (24.8%)	
PI, Med (IQR)	25.215 (7.392–48.990)	23.070 (5.950–44.210)	0.205

### 3.2 Identification of baseline characteristics significantly affecting patient OS

A univariate and a multivariate Cox regression model were used to identify the baseline characteristics significantly affecting patient OS. After performing multivariate Cox regression, the *p*-values were adjusted using the Benjamini–Hochberg correction to control the false positive rate (Palomino-Fernández et al., 2024). As shown in Table 3, ECOG (Hazard Ratio (HR) = 0.481, 95% Confidence Interval (CI): 0.372–0.623, *p*-value <0.001), EOD (HR = 2.084, 95% CI: 1.581–2.749, *p*-value <0.001) and PI (HR = 1.005, 95% CI: 1.002–1.009, *p*-value = 0.006) were important factors affecting OS in the univariate cox regression, after adjust for other characteristics, these three variables were still associated with OS (ECOG (HR = 0.521, 95% CI: 0.399–0.680, adjusted *p*-value <0.001), EOD (HR = 2.054, 95% CI: 1.557–2.709, adjusted *p*-value <0.001), PI (HR = 1.004, 95% CI: 0.999–1.008, adjusted *p*-value = 0.021)).

**TABLE 3:** Results of univariate Cox regression and multivariate Cox regression.

Variable	Univariate analysis			Multivariable analysis		
	HR	95% CI	*p*-value	HR	95% CI	Adjusted *p*-value
Age	1.006	(0.996–1.017)	0.318	—	—	—
Sex	1.081	(0.868–1.348)	0.486	—	—	—
ECOG	0.481	(0.372–0.623)	<0.001***	0.521	(0.399–0.680)	<0.001***
EOD	2.084	(1.581–2.749)	<0.001***	2.054	(1.557–2.709)	<0.001***
PI	1.005	(1.002–1.009)	0.006**	1.004	(0.999–1.008)	0.021*

### 3.3 Comparison of the efficacy of GEM-E and GEM treatment regimens

**FIGURE 1:**
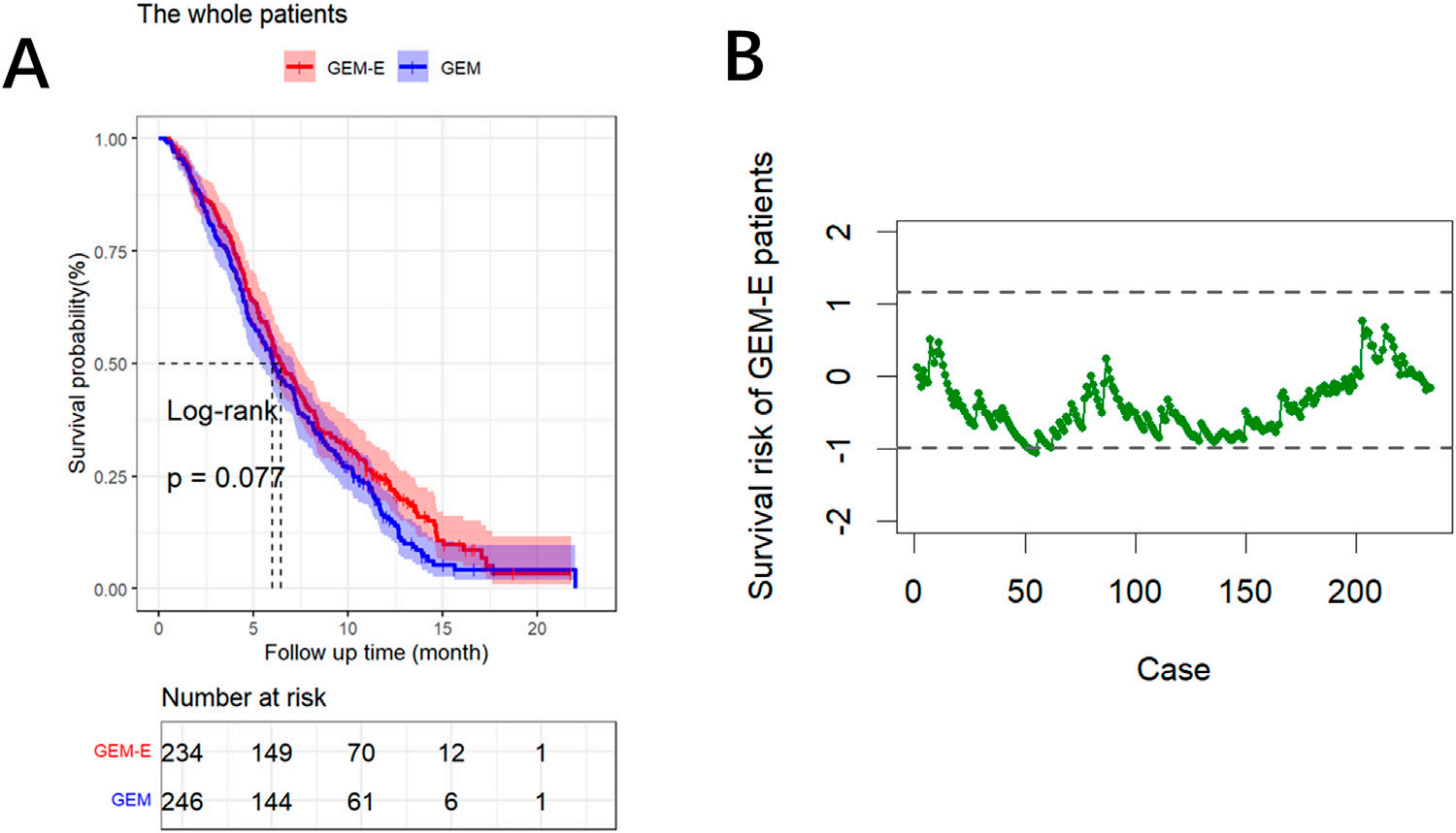
Results of the comparison. **(A)** Survival curves comparing the GEM-E and GEM treatments. **(B)** RAES chart comparing the GEM-E and GEM treatments.

We compared the survival outcomes of PDAC patients under the two treatment regimens using Kaplan-Meier survival curve analysis. As shown in Figure 1A, the overall survival in the GEM-E group was not significantly prolonged (*p*-value = 0.077). Furthermore, to account for patient heterogeneity, we used the RAES chart to assess the survival performance of patients treated with GEM-E based on the survival performance of patients treated with GEM. Figure 1B showed the chart statistics of the patients accepting GEM-E therapy based on the weighted score test, the chart statistics used to measure the patient’s survival risk. Specifically, there would be a significant improvement in GEM-E assignment when the chart statistic was below $L_{l}$ and a significant deterioration when the chart statistic was upper $L_{u}$ . It could be concluded that the majority of GEM-E patients were within the upper and lower control limits except the 53, 55 and 62 cases exceeding the lower control limits and triggering alarms. This suggested that although there was no significant improvement in overall survival, three case showed significant improvement, highlighting the heterogeneity in treatment exist.

### 3.4 Identification of predictive biomarkers

**FIGURE 2:**
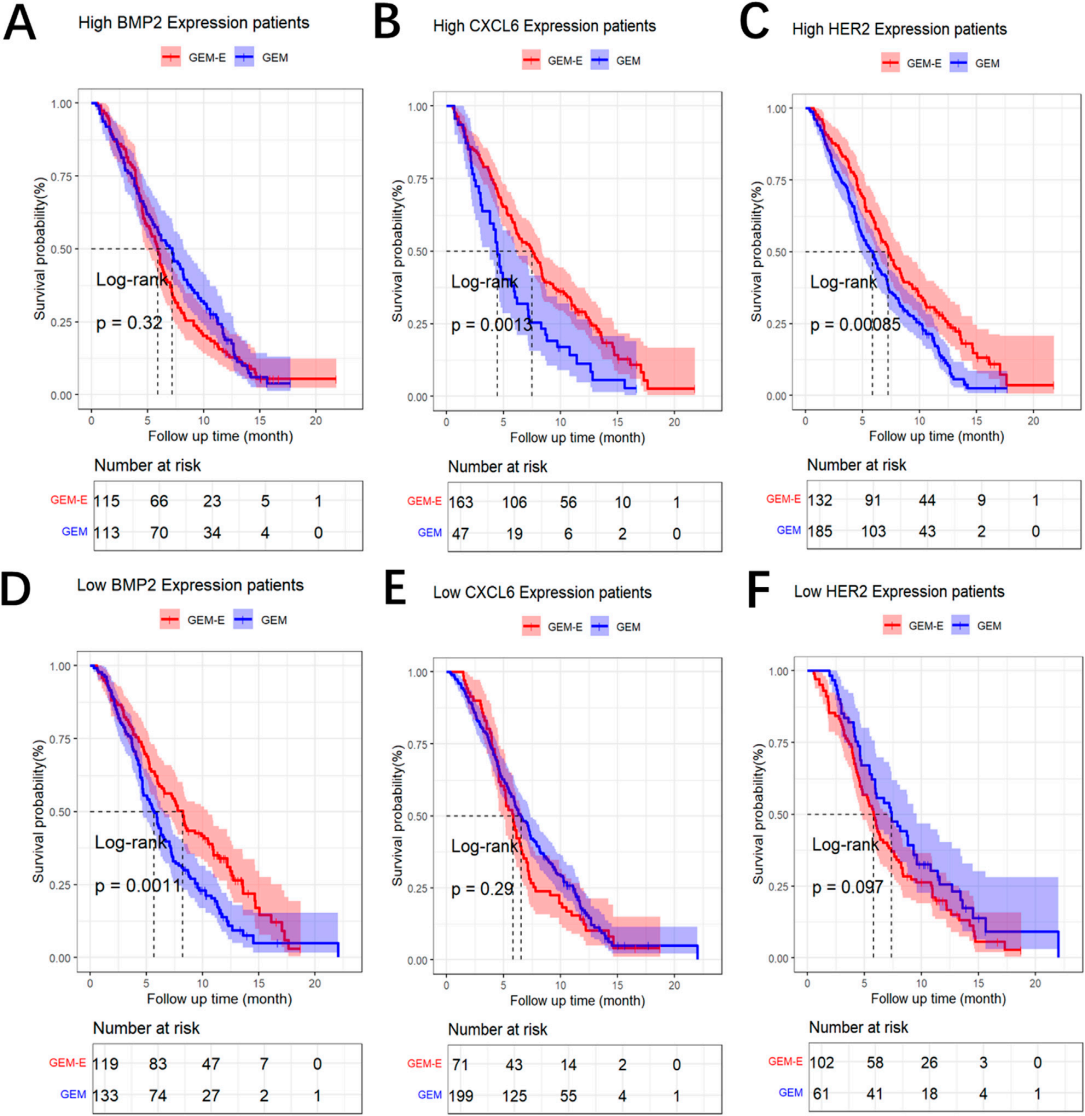
Results of the comparison. **(A)** Log-rank Survival Analysis of GEM vs. GEM-E Efficacy by high BMP2 Expression Level. **(B)** Log-rank Survival Analysis of GEM vs. GEM-E Efficacy by high CXCL6 Expression Level. **(C)** Log-rank Survival Analysis of GEM vs. GEM-E Efficacy by high HER2 Expression Level. **(D)** Log-rank Survival Analysis of GEM vs. GEM-E Efficacy by low BMP2 Expression Level. **(E)** Log-rank Survival Analysis of GEM vs. GEM-E Efficacy by low CXCL6 Expression Level. **(F)** Log-rank Survival Analysis of GEM vs. GEM-E Efficacy by low HER2 Expression Level.

We constructed maximally selected rank statistics to determine the optimal cut-point for each biomarker, and then identified predictive biomarkers that exhibited a significant interaction effect between GEM-E treatment and the binary biomarker, with the findings provided in Supplementary Figure S1 of the Supplementary Material A2. To overcome overfitting, we employ the internal bootstrap method to assess the stability of the cut-point (He et al., 2018; Santagata et al., 2024). For each bootstrap sample obtained from resampling with replacement from the data for all 480 patients, the patients were divided into two subsets based on the cut-point. Subsequently, a conventional proportional hazards model was fitted using the interaction term between the binary biomarker and treatment. In 1,000 bootstrap samples, the results exhibiting a significant interaction between the binary HER2, CXCL6, BMP2 and treatment with a proportion of 90.6%, 88.7% and 85.2%, respectively. These findings suggest that the cut-point is stable. As shown in Figure 2, patients with BMP2 levels lower than 103,836 had improved survival when treated with GEM-E (median OS: 8.214 vs. 5.684 months, HR: 0.630, 95% CI: 0.477–0.832, *p*-value: 0.001**) whereas no significant difference between GEM-E and GEM was found from patients with BMP2 levels above than 103,836 (median OS: 5.914 vs. 7.162 months, HR: 1.149, 95% CI: 0.873–1.512, *p*-value: 0.320) (Figures 2A, D); Patients with CXCL6 levels above than 4,08,510 had improved survival when treated with GEM-E (median OS: 7.491 vs. 4.468 months, HR: 0.572, 95% CI: 0.405–0.807, *p*-value: 0.001**) whereas no significant difference between GEM-E and GEM was found from patients with CXCL6 levels lower than 4,08,510 (median OS: 5.815 vs. 6.538 months, HR: 1.169, 95% CI: 0.879–1.555, *p*-value: 0.290) (Figures 2B, E); Patients with HER2 levels above than 6,718 had improved survival when treated with GEM-E (median OS: 7.228 vs. 5.881 months, HR: 0.659, 95% CI: 0.515–0.844, *p*-value <0.001***) whereas no significant difference between GEM-E and GEM was found from patients with HER2 levels lower than 6,718 (median OS: 5.799 vs. 7.359 months, HR: 1.341, 95% CI: 0.946–1.900, *p*-value: 0.097) (Figures 2C, F). We have added the Kaplan-Meier curve stratify for the biomarker-class within the two treatment groups, the results also demonstrate the predictive capabilities of these three biomarkers, with the findings provided in Supplementary Figure S2 of the Supplementary Material A3. In summary, HER2, CXCL6, and BMP2 were identified as potential predictive biomarkers for GEM-E in our study.

Although our study is a correlative study linking the biomarker measures with clinical outcomes and there was no experimental and functional studies to validate the biological mechanisms and substantiate that the predictive value of identified biomarkers (BMP2, CXCL6, and HER2), we conducted a literature review and found relevant experimental and functional studies that illustrate the role of these biomarkers. HER2 is a transmembrane tyrosine kinase receptor that belongs to the EGFR family. By activating multiple downstream signaling pathways, such as PI3K/AKT, RAS/RAF/MEK/ERK, and JAK/STAT pathways, HER2 promotes cell proliferation, survival, and metabolic regulation, while also enhancing tumor cell migration and invasiveness. These functions enable HER2 to play a critical role in tumor initiation, progression, and metastasis. The overactivation of HER2 not only leads to uncontrolled proliferation and resistance to apoptosis in tumor cells but also increases the tumor’s resistance and metastatic potential, making the cancer more difficult to treat (Moasser, 2007). HER2 was identified as a potential predictive biomarker for GEM-E in Shultz et al. (2016), suggesting that we could use HER2 to select patients most likely to respond to GEM-E. In addition, we found two other predictive biomarkers that were not detected by Shultz et al. (2016). CXCL6 was identified as a potential predictive biomarker for hepatocellular carcinoma patients undergoing trans arterial chemoembolization in Kinzler et al. (2023); CXCL6 is an inflammatory chemokine that plays a crucial role in immune response and cancer progression. It promotes tumor growth and enhances metastasis by recruiting immune cells to the tumor microenvironment. CXCL6 exerts its effects by interacting with its main receptors, CXCR1 and CXCR2, activating downstream signaling pathways that regulate cell migration, invasion, and survival. In cancer, CXCL6 is often upregulated, and its expression is associated with poor prognosis in various malignancies, including colorectal cancer, gastric cancer, and others. Additionally, CXCL6, together with other immune modulators such as CXCL8 and IL-8, contributes to the formation of an immunosuppressive microenvironment that facilitates tumor escape from immune surveillance, thereby promoting cancer progression and resistance to therapy (Fernandez-Avila et al., 2023). BMP2 was identified as a predictive biomarker for patients with advanced non-small cell lung cancer undergoing chemotherapy in Fei et al. (2013). BMP2 is a member of the transforming growth factor-beta (TGF-β) superfamily and is implicated in embryonic development and postnatal homeostasis in tissues and organs. Experimental research in the contexts of physiology and pathology has indicated that BMP2 can induce macrophages to differentiate into osteoclasts and accelerate the osteolytic mechanism, aggravating cancer cell bone metastasis (Li et al., 2022). The potential predictive roles of HER2, CXCL6 and BMP2 as biomarkers in the treatment of different cancers had suggested that they may have played significant roles in cancer biology. Extending the research on these biomarkers to the context of PDAC patients who received GEM-E combination therapy held significant implications.

However, we assumed that the patients in the same subgroup enjoy the same treatment effect, there may be heterogeneity even within the same subgroup due to unobserved factors. We tried to use the RAES chart to assess the survival performance of patients treated with GEM-E based on the survival performance of patients treated with GEM in each subgroup.

### 3.5 Identification of treatment-sensitive in each subgroup

**FIGURE 3:**
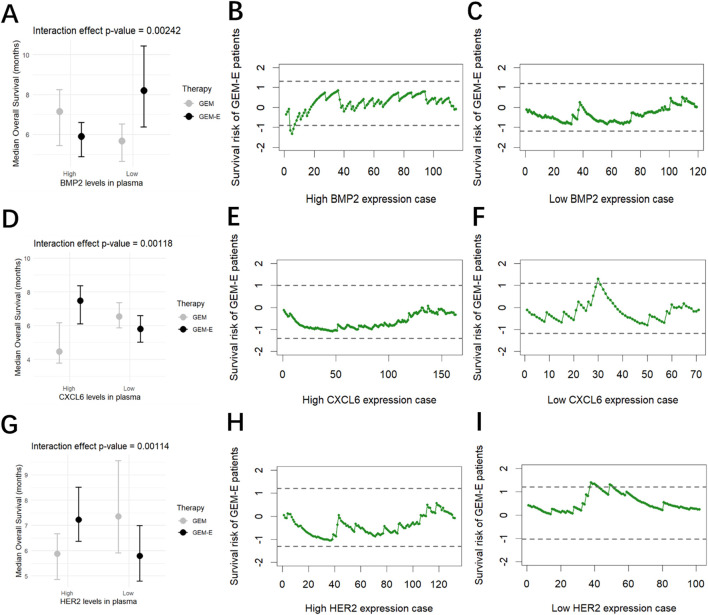
Results of the comparison. **(A)** Interaction of BMP2 levels with Therapy. **(B)** Survival risk of patients with high BMP2 levels treated with GEM-E. **(C)** Survival risk of patients with low BMP2 levels treated with GEM-E. **(D)** Interaction of CXCL6 levels with Therapy. **(E)** Survival risk of patients with high CXCL6 levels treated with GEM-E. **(F)** Survival risk of patients with low CXCL6 levels treated with GEM-E. **(G)** Interaction of HER2 levels with Therapy. **(H)** Survival risk of patients with high HER2 levels treated with GEM-E. **(I)** Survival risk of patients with low HER2 levels treated with GEM-E.

For each of the predictive biomarkers what we identified in the previous section (BMP2, CXCL6 and HER2), we grouped patients into high-expression level groups and low-expression level groups, and then used RAES charts to monitor the survival risk of patients receiving GEM-E therapy compared to those receiving GEM therapy. The monitoring results were shown in Figure 3. When BMP2 was highly expressed, the majority of GEM-E patients were within the upper and lower control limits, except for the 4, 5, and 6 cases that exceeded the lower control limits and triggered an alarm. This suggested that although there was no significant improvement in overall survival, three cases showed a significant improvement (Figures 3A–C). When CXCL6 was lower expressed, the survival risk of patients under the GEM-E therapy was generally within the upper and lower control limits, with case 30 exceeding above the upper control limit (Figures 3D–F). When HER2 was lower expressed, the survival risk of patients under the GEM-E therapy was generally higher compared to the GEM therapy, with cases 38, 39, 40, 41, 42, 49, and 50 exceeding above the upper control limit (Figures 3G–I). For other groups, such as those with low BMP2 expression, high CXCL6 expression, and high HER2 expression, the majority of the survival risk of GEM-E patients was negative, suggesting that there was an improvement in overall survival when they accepted GEM-E. The conclusion was consistent with the forest plot in Figure 3. The RAES control charts not only validated the predictive biomarkers but also identified the treatment-sensitive patients in each subgroup. Further research could analyze the reasons for their sensitivity, which may have helped healthcare professionals to develop more targeted and effective treatment strategies.

### 3.6 Sensitivity analysis

To test the sensitivity of the three variables (ECOG performance status (ECOG), extent of disease (EOD), and pain intensity (PI)), we divided the dataset into different subgroups based on Sex (0, female vs. 1, male) and Age (≤65 vs. >65) (Hammel et al., 2016), and conducted univariate and multivariate Cox regression analysis within each subgroup to confirm the independence and robustness of the key variables in the model. The results are shown in Supplementary Tables S2, S3 of the Supplementary Materials A4, where the three variables are significantly associated with patient prognosis in each subgroup, confirming the robustness of the findings.

## 4 Discussion

PDAC is a highly invasive and poor-prognosis malignant tumor (Halbrook et al., 2023). Traditional chemotherapy drugs like GEM constitute the cornerstone of PDAC treatment. With advances in medicine, combination therapies such as GEM-E have been proposed to enhance treatment efficacy. However, if combination therapy does not significantly improve objective response rates over monotherapy and is accompanied by side effects, reassessment of the treatment regimen is necessary to ensure patients receive the most effective treatment while minimizing unnecessary risks and burdens. Additionally, considering that clinical trials are typically conducted under stringent conditions whereas real-world patient populations are more complex and diverse, any treatment regimen showing promise in clinical trials requires further validation of its effectiveness and safety in real clinical settings. This is particularly crucial for PDAC due to its high heterogeneity, where different patients may respond very differently to treatment. On the other hand, with the popularization of precision medicine and personalized treatment concepts, research into tumor biomarkers has become increasingly important. Certain biomarkers may indicate a patient’s sensitivity or resistance to specific drugs, thereby guiding clinical decisions. Modern medical technologies such as gene sequencing, proteomics, and metabolomics provide powerful tools for identifying and validating biomarkers (Dar et al., 2023). With the development of big data and artificial intelligence technologies, clinical data can be analyzed more effectively to identify and validate biomarkers predictive of treatment efficacy, thus offering more personalized treatment options for patients.

The aim of this study was to use data from the PA.3 trial to compare the actual treatment effects of GEM-E and GEM treatments in the context of patient heterogeneity and to identify predictive biomarkers. Beyond this specific context, this method can be adapted to other clinical trails with biomarkers to find the treatment-sensitive subgroups. Firstly, univariate and multivariate Cox proportional hazards models were used to identify baseline characteristics associated with OS. Then, a risk-adjusted control chart based on a weighted score test from the Cox model was constructed to monitor patients’ survival risk. Next, maximally selected rank statistics were constructed to identifying the predictive biomarkers. Finally, a risk adjusted control chart based on a weighted score test from the Cox model was constructed to validate the predictive biomarkers to discover the patients who sensitive to the GEM-E. These findings help healthcare providers offer more precise treatment strategies for PDAC patients, thereby providing a reference for improving patient survival outcomes.

Our study found that BMP2, CXCL6, and HER2 serve as predictive biomarkers for GEM-E, suggesting that we can use them to select patients most likely to respond to GEM-E. The following are the steps for the Application of these findings to clinical practice: Prior to treatment, measure the levels of the three biomarkers (BMP2, CXCL6, and HER2) in patients. Based on the threshold values determined in this trial, patients with low BMP2 levels, high CXCL6 levels, or high HER2 levels are more likely to respond to combination therapy, thereby recommending combination therapy for these patients. Prior to this, Shultz et al. (2016) identified HER2 as a potential predictive biomarker for GEM-E, Kinzler et al. (2023) identified CXCL6 as a potential predictive biomarker for hepatocellular carcinoma patients undergoing trans arterial chemoembolization, and Fei et al. (2013) identified BMP2 as a predictive biomarker for patients with advanced non-small cell lung cancer undergoing chemotherapy. The potential predictive roles of these biomarkers in the treatment of different cancers indicate that they play significant roles in cancer biology. Our study also identified populations sensitive to GEM-E therapy. Specifically, in cases where the effects of the two therapies did not significantly differ (such as high BMP2 expression cases, low CXCL6 expression cases, and low HER2 expression cases), we had discovered patients sensitive to GEM-E therapy based on predictive biomarkers. Identifying sensitive cases helps healthcare professionals analyze the underlying reasons for their sensitivity, thereby enabling the development of more targeted and effective treatment strategies.

However, our study has several limitations. Firstly, this study is prospective–retrospective study based on data from a single clinical trial. Therefore, the results may be applicable only to patients who satisfy the eligibility criteria of the trial and receive similar treatments. The method used in this paper could be used, however, to identify predictive biomarkers from data in other trials when the data on biomarkers are available. The original design of the trial was only powered to detect the difference between two treatment groups and may not be able to identify some important subgroups. Some important confounding variables, such as genetic factors, lifestyle, and environmental factors (Maisonneuve and Lowenfels, 2015), were not collected from the trial and, therefore, could not be adjusted, which may make our conclusions less reliable. Secondly, the study only utilized expression data from 15 biomarkers, which may not fully reflect the complexity of PDAC and may miss other important predictive factors. In addition, the proposed approach is based solely on data from the patients in this study, this makes the suggested cutoff values unable to be thoroughly validated in different clinical settings. Finally, the study could not consider possible variations in biomarker levels over time throughout the treatment process since only baseline measurements of biomarkers were available. If the biomarker levels over time are avaiable, we could utilize quality control measures to monitor the changes in biomarker levels in real time and provide early warnings for any abnormal fluctuations, thereby preventing the deterioration of the patient’s condition in advance. Finally, The expected false alarm rate directly influences the setting of the upper and lower control limits. To balance Type I and Type II errors, we selected an optimal empirical value for the false alarm rate based on existing research (Lai et al., 2021). However, although this empirical value effectively controls both types of errors in practice, there is a certain discrepancy between it and the theoretical value. Therefore, although this empirical value is useful in practice, it may not fully reflect all real-world situations in different datasets or control environments.

In summary, our study demonstrated that GEM-E treatment for pancreatic cancer did not show significant superiority over GEM in reality, with only moderate differences in survival observed between the two. We identified several predictive biomarkers, and these results were consistent with previous studies. Additionally, we used control chart methods to identify population types sensitive to the treatment regimens based on the results of the maximally selected rank statistics. These findings provide hope for individuals who may derive greater benefits from either treatment regimen.

## Data Availability

The data analyzed in this study is subject to the following licenses/restrictions: All data included in this study are available upon request by contact with the authors. Requests to access these datasets should be directed to DT, dtu@ctg.queensu.ca.
