# Supplementary material for: Identification and validation the predictive biomarkers based on risk-adjusted control chart in gemcitabine with or without erlotinib for pancreatic cancer therapy
Source: Front Genet. 2024 Dec 17;15:1497254. doi: 10.3389/fgene.2024.1497254 (PMC11685217; doi:10.3389/fgene.2024.1497254)
Supplement: Supplementary file 1 [file Table1.docx]

Supplementary to “Identification and validation the predictive biomarkers based on risk-adjusted control chart in Gemcitabine with or without Erlotinib for Pancreatic Cancer Therapy”

Aijun Zhao^1^, Dongsheng Tu^2^, Ye He^3^, Liu Liu^1^, Bin Wu^4*^, Yixing Ren^5*^

^1^College of Mathematics and Physics and Geomathematics Key Laboratory of Sichuan Province, Chengdu University of Technology, Chengdu, China

^2^Department of Public Health Sciences, Canadian Cancer Trials Group, Queen's University, Kingston, Ontario, Canada

^3^Visual Computing and Virtual Reality Key Laboratory of Sichuan Province, Sichuan Normal University, Chengdu, China

^4^North Sichuan Medical College, Nanchong, China

^5^Department of General Surgery, and Institute of Hepato-Biliary-Pancreas and Intestinal Disease, Affiliated Hospital of North Sichuan Medical College, Nanchong, China

*** Correspondence:**Bin Wu: 893625234@qq.com
Yixing Ren: yixingren@nsmc.edu.cn

**Contents**

[**A1. The Results of the Schoenfeld residual test 2**](#_Toc183096002)

[**A2. The Results of the identification of the predictive biomarkers 3**](#_Toc183096003)

[**A3. The Results of the Kaplan-Meier curves 4**](#_Toc183096004)

[**A4. The Results of the Sensitivity analyses 5**](#_Toc183096005)

# A1. The Results of the Schoenfeld residual test

Table S1: The result of the Schoenfeld residual test

| Variable |  | Univariate analysis | |  | Multivariable analysis | | |  |
| --- | --- | --- | --- | --- | --- | --- | --- | --- |
|  |  | chisq | p-value |  | chisq | p-value | |  |
| Age |  | 2.583 | 0.108 |  | — | | — |  |
| Sex |  | 3.122 | 0.077 |  | — | | — |  |
| ECOG |  | 0.685 | 0.408 |  | 0.682 | | 0.409 |  |
| EOD |  | 3.328 | 0.068 |  | 3.308 | | 0.069 |  |
| PI | |  | 1.113 | 0.291 |  | 1.293 | | 0.256 |

chisq: chi-square statistic.

# A2. The Results of the identification of the predictive biomarkers


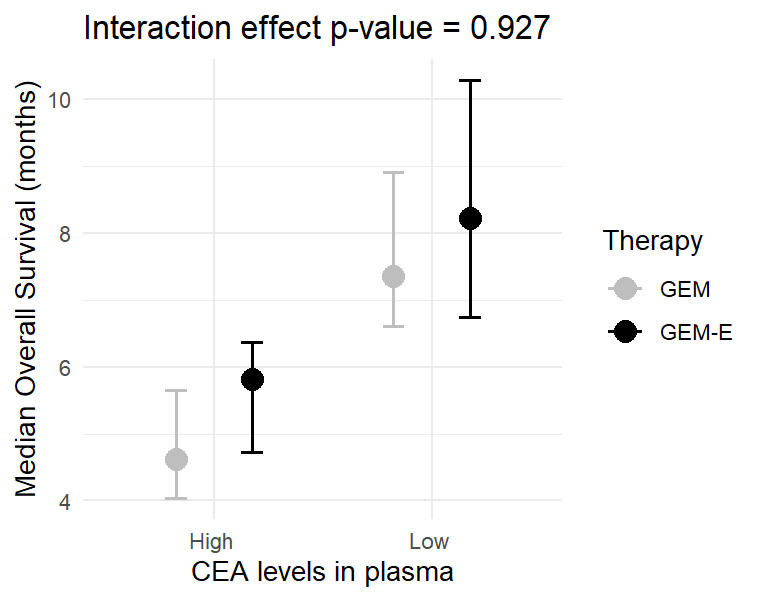

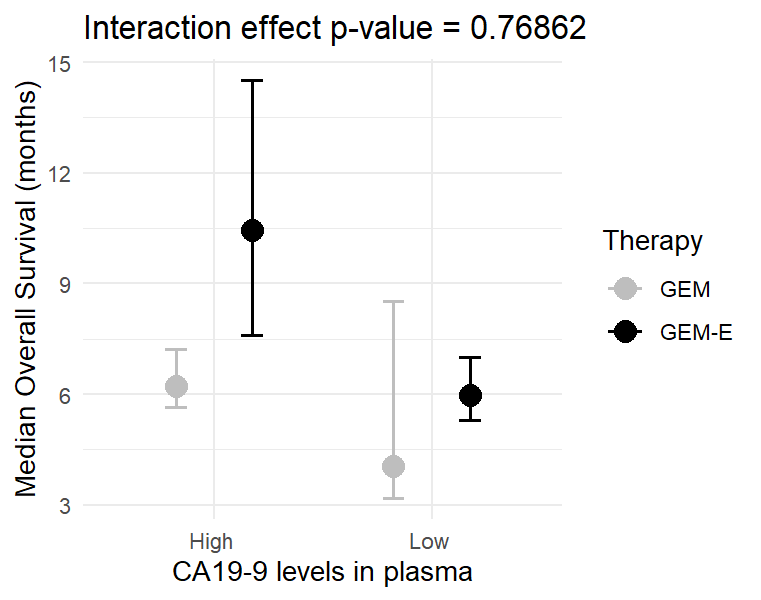

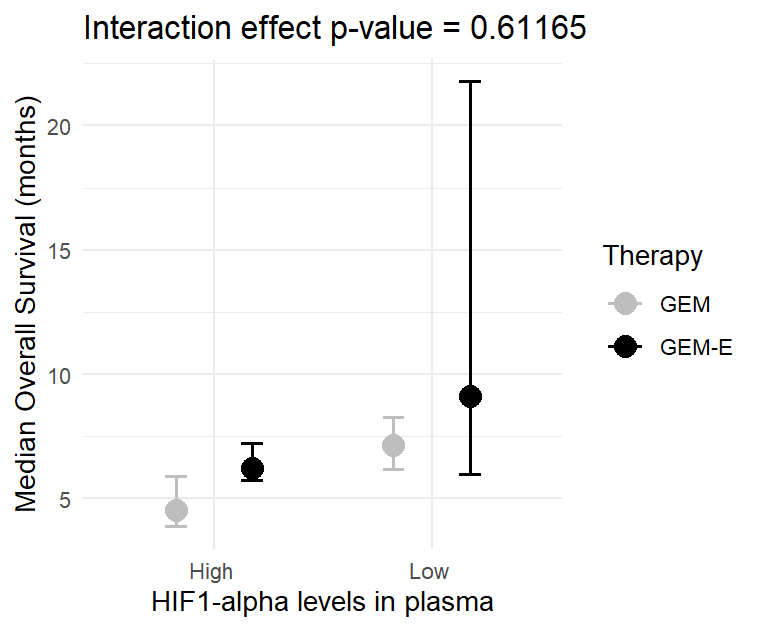

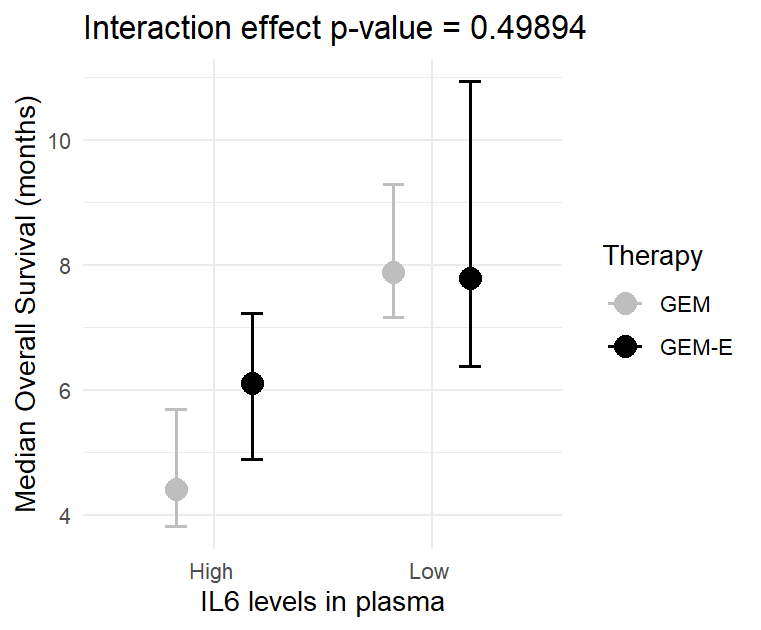

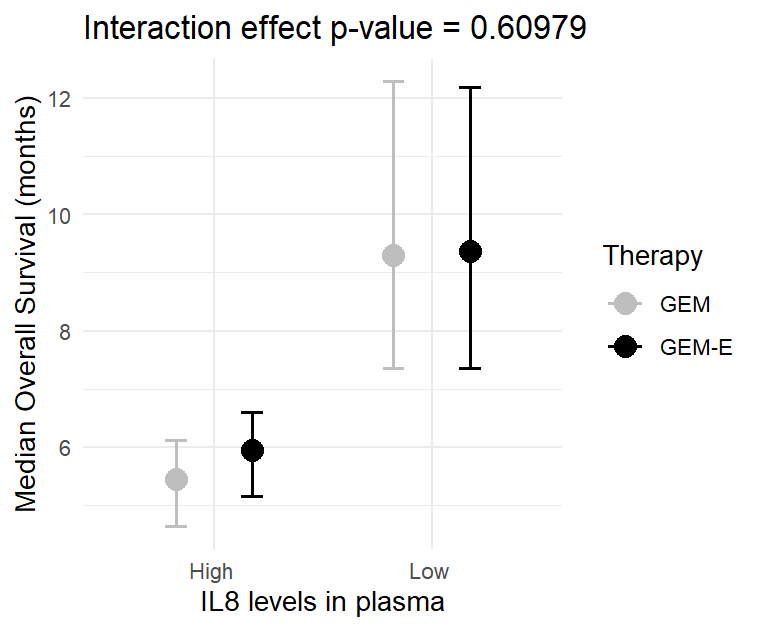

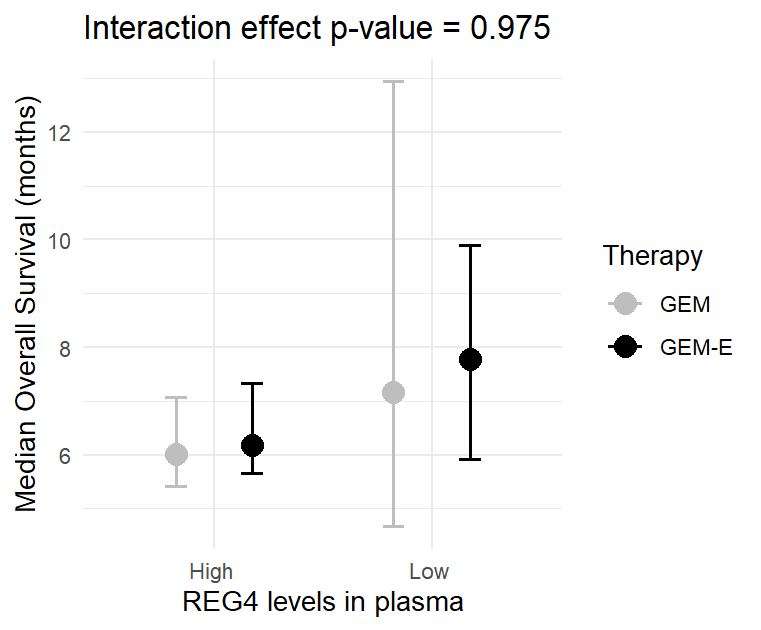

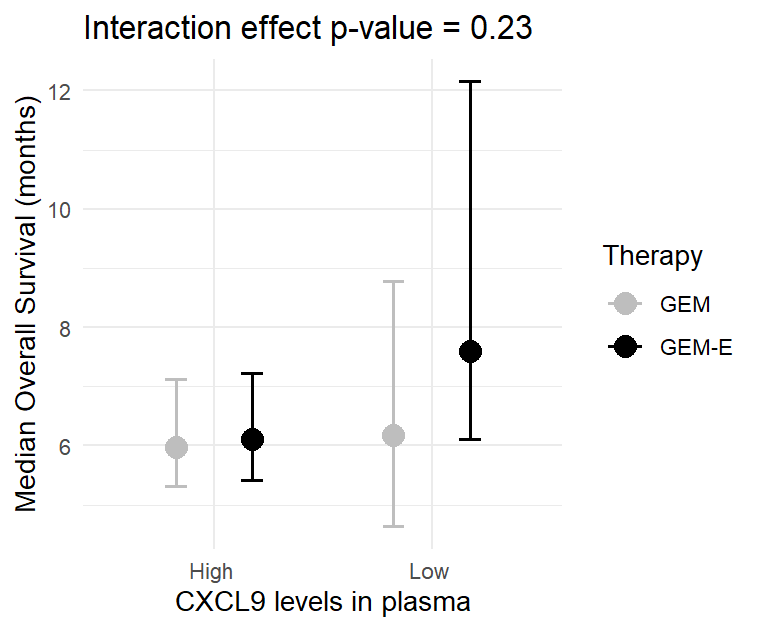

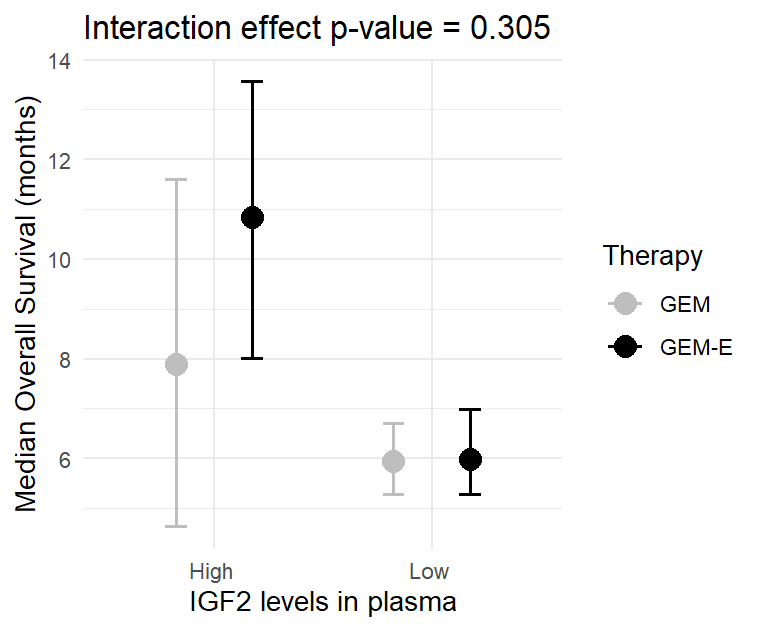

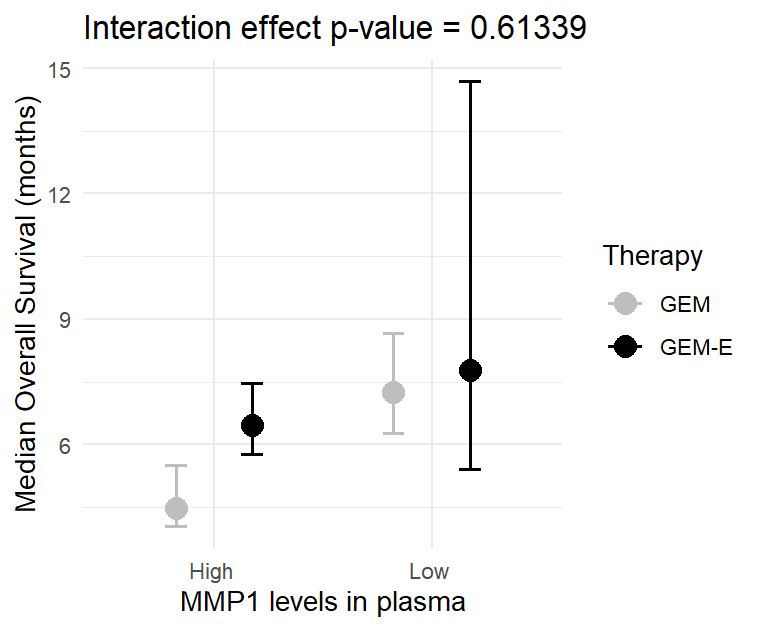

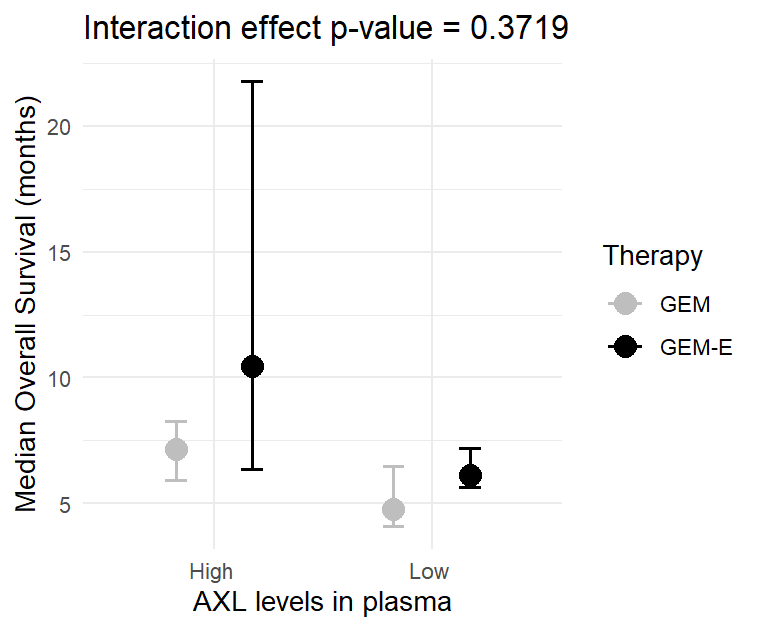

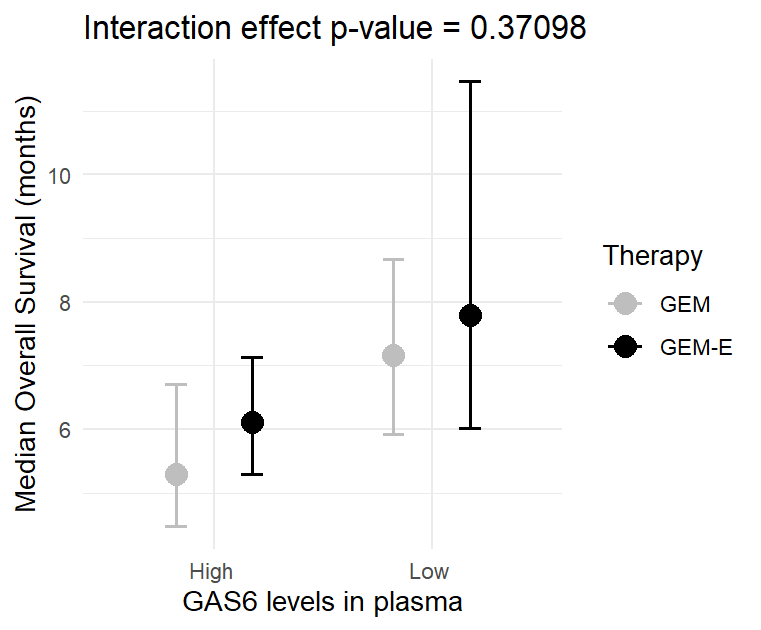

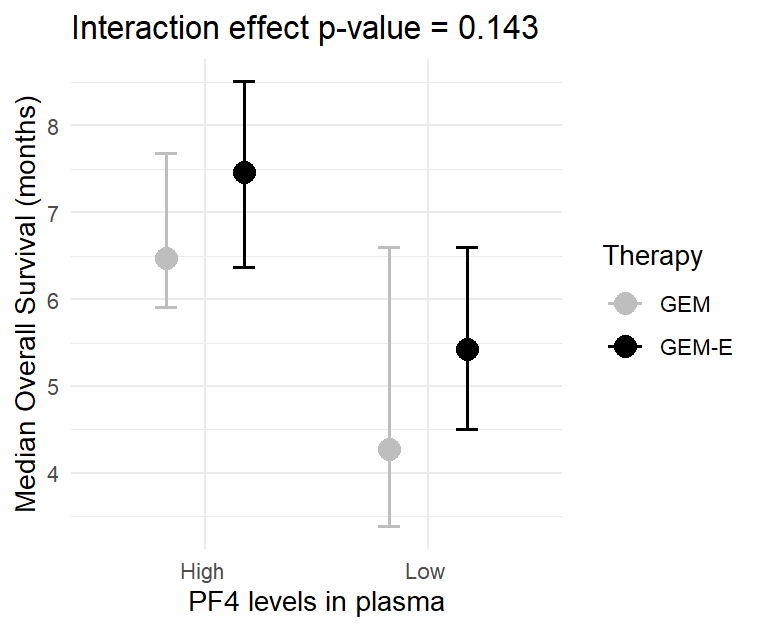


Figure S1: Interaction of CEA, CA19-9, HIF1-alpha, IL6, IGF2, MMP1, CXCL9, REG4, AXL, IL8, PF4, GAS6 levels with Therapy.

# A3. The Results of the Kaplan-Meier curves


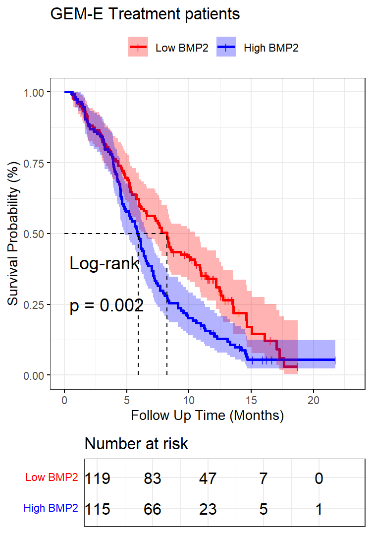

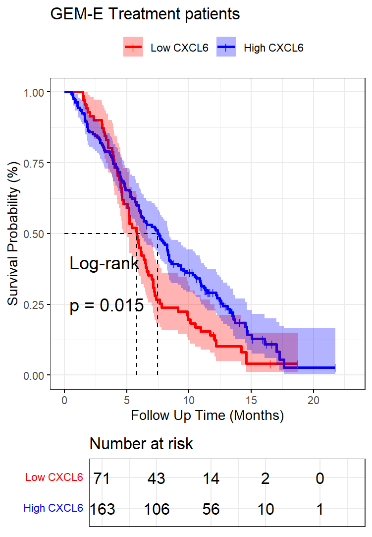

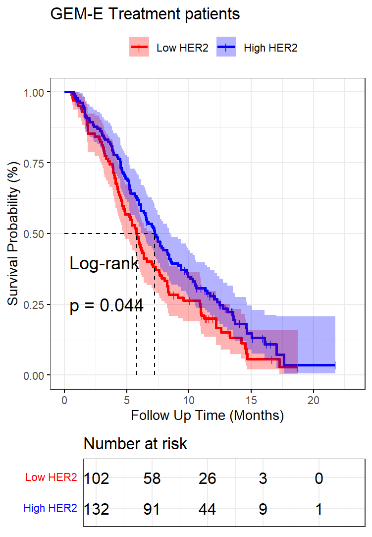

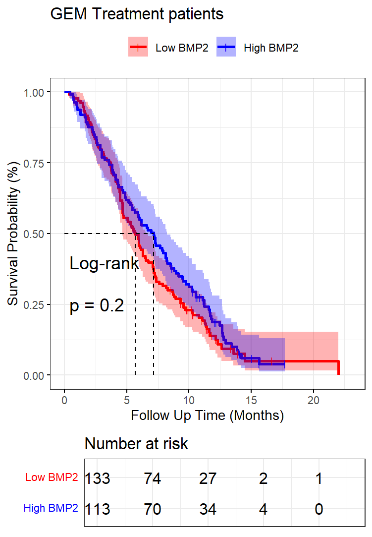

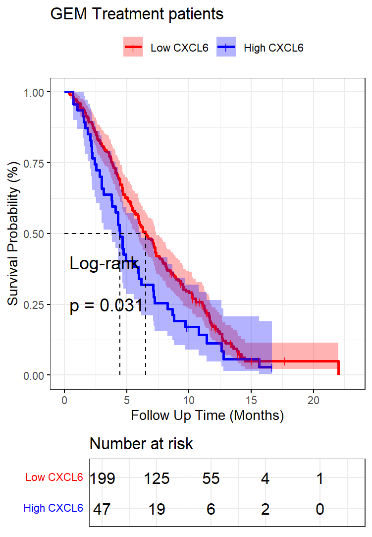

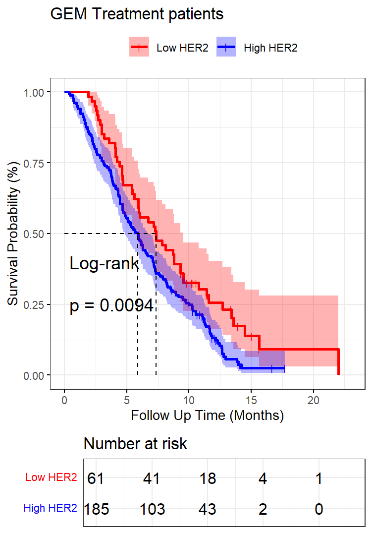


Figure S2: The Kaplan -Meier curve stratify for the biomarker-class within the two treatment groups.

# A4. The Results of the Sensitivity analyses

Table S2: Results of univariate Cox regression and multivariate Cox regression based on sex

| Variable |  | Univariate analysis | | |  | Multivariable analysis | | | |
| --- | --- | --- | --- | --- | --- | --- | --- | --- | --- |
|  |  | HR | 95% CI | p-value |  | HR | | 95% CI | Adjusted p-value |
| The female patients | | | | | | | | | |
| Age |  | 1.005 | (0.991-1.020) | 0.463 |  | — | — | | — |
| ECOG |  | 0.439 | (0.308-0.625) | < 0.001*** |  | 0.517 | (0.358-0.749) | | < 0.001*** |
| EOD |  | 2.305 | (1.602-3.315) | < 0.001*** |  | 2.342 | (1.621-3.382) | | < 0.001*** |
| PI |  | 1.007 | (1.002-1.013) | 0.007** |  | 1.006 | (1.001-1.012) | | 0.022* |
| The male patients | | | | | | | | | |
| Age |  | 1.005 | (0.989-1.022) | 0.514 |  | — | — | | — |
| ECOG |  | 0.542 | (0.371-0.795) | 0.002** |  | 0.551 | (0.373-0.815) | | 0.003** |
| EOD |  | 1.839 | (1.197-2.825) | 0.005** |  | 1.829 | (1.190-2.812) | | 0.006** |
| PI |  | 1.013 | (1.007-1.017) | < 0.001*** |  | 1.018 | (1.008-1.028) | | 0.002** |

Table S3: Results of univariate Cox regression and multivariate Cox regression based on Age

| Variable |  | Univariate analysis | | |  | Multivariable analysis | | | |
| --- | --- | --- | --- | --- | --- | --- | --- | --- | --- |
|  |  | HR | 95% CI | p-value |  | HR | | 95% CI | Adjusted p-value |
| The patients with Age≤65 | | | | | | | | | |
| Sex |  | 1.153 | (0.843-1.577) | 0.371 |  | — | — | | — |
| ECOG |  | 0.469 | (0.330-0.668) | < 0.001*** |  | 0.519 | (0.362-0.745) | | < 0.001*** |
| EOD |  | 2.210 | (1.485-3.289) | < 0.001*** |  | 2.343 | (1.571-3.494) | | < 0.001*** |
| PI |  | 1.010 | (1.004-1.015) | < 0.001*** |  | 1.008 | (1.003-1.014) | | < 0.001*** |
| The patients with Age > 65 | | | | | | | | | |
| Sex |  | 1.058 | (0.771-1.451) | 0.727 |  | — | — | | — |
| ECOG |  | 0.501 | (0.342-0.735) | < 0.001*** |  | 0.501 | (0.334-0.750) | | < 0.001*** |
| EOD |  | 1.961 | (1.330-2.889) | < 0.001*** |  | 1.894 | (1.284-2.795) | | 0.001** |
| PI |  | 1.005 | (1.001-1.011) | <0.001*** |  | 1.010 | (1.004-1.017) | | < 0.001*** |
